# The UK Standing Dental Advisory Committee (1948-2010) with special reference to its Expert Working Party on Orthodontics, 1992

**DOI:** 10.1038/s41415-025-9200-7

**Published:** 2026-02-13

**Authors:** Christopher Stephens

**Affiliations:** Emeritus Professor of Child Dental Health, University of Bristol, Bristol, United Kingdom

## Abstract

While the British Dental Association initially advised its members against joining the NHS (National Health Service), its success for dentistry was assured by the enthusiastic support of the Incorporated Dental Society Ltd, led by Frederick Ballard. Regarded by the Socialist Medical Association, of which he was a member, as the ‘voice of British dentistry', Ballard was one of the first members of the Central Health Services Council set up in 1946 to provide advice to the Government. Subsequently, the Standing Dental Advisory Committee – one of four which Aneurin Bevan set up in 1949 – continued to provide valuable professional advice to successive Ministers of Health until it was abolished by Alan Johnson in 2010.

## Key points

- Provides a history of the development of the dental advisory input to the UK health service.
- Describes how the Working Party on Orthodontics in the health service came to be set up in 1991.
- Shows that the discussions within the Working Party brought about changes in the delivery and improvement in the standard of UK orthodontic treatment.
- Suggests that the abolition of the Standing Dental Advisory Committee may have led to a deterioration in the communication between the dental profession and the Government.

## Introduction

When the National Health Service (NHS) was set up, provision was made in the 1946 NHS Act for there to be a Central Health Services Council to advise the Ministry of Health, which also had direct access to the Minister.^1^ There were three dental practitioners on the Central Council.^2^ One of the first to serve on both this and later, the Standing Dental Advisory Committee (SDAC), was Frederick Ballard OBE, who had done so much to ensure that dentistry, including orthodontic treatment, was an integral part of the NHS.^3^

The period after the Second World War was one of very rapid change and to deal with this, Parliament passed the Statutory Instruments Act.^4^ This established the concept of ‘secondary legislation' which allowed Parliament to include specific delegated powers to ministers within any subsequent Acts it passed. Parliament's role in secondary legislation only involved scrutinising and approving such measures rather than requiring parliamentary time to introduce a new Act. In extreme urgency, Statutory Instruments could be approved by the Privy Council alone.

**Fig. 1:**
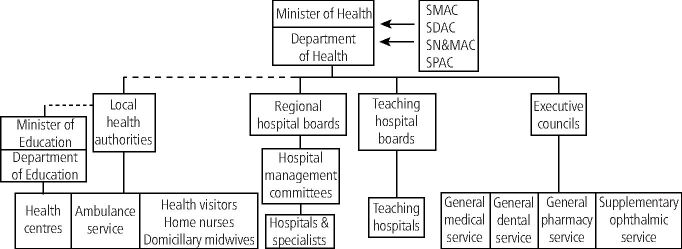
The structure of the NHS in 1949

**Fig. 2:**
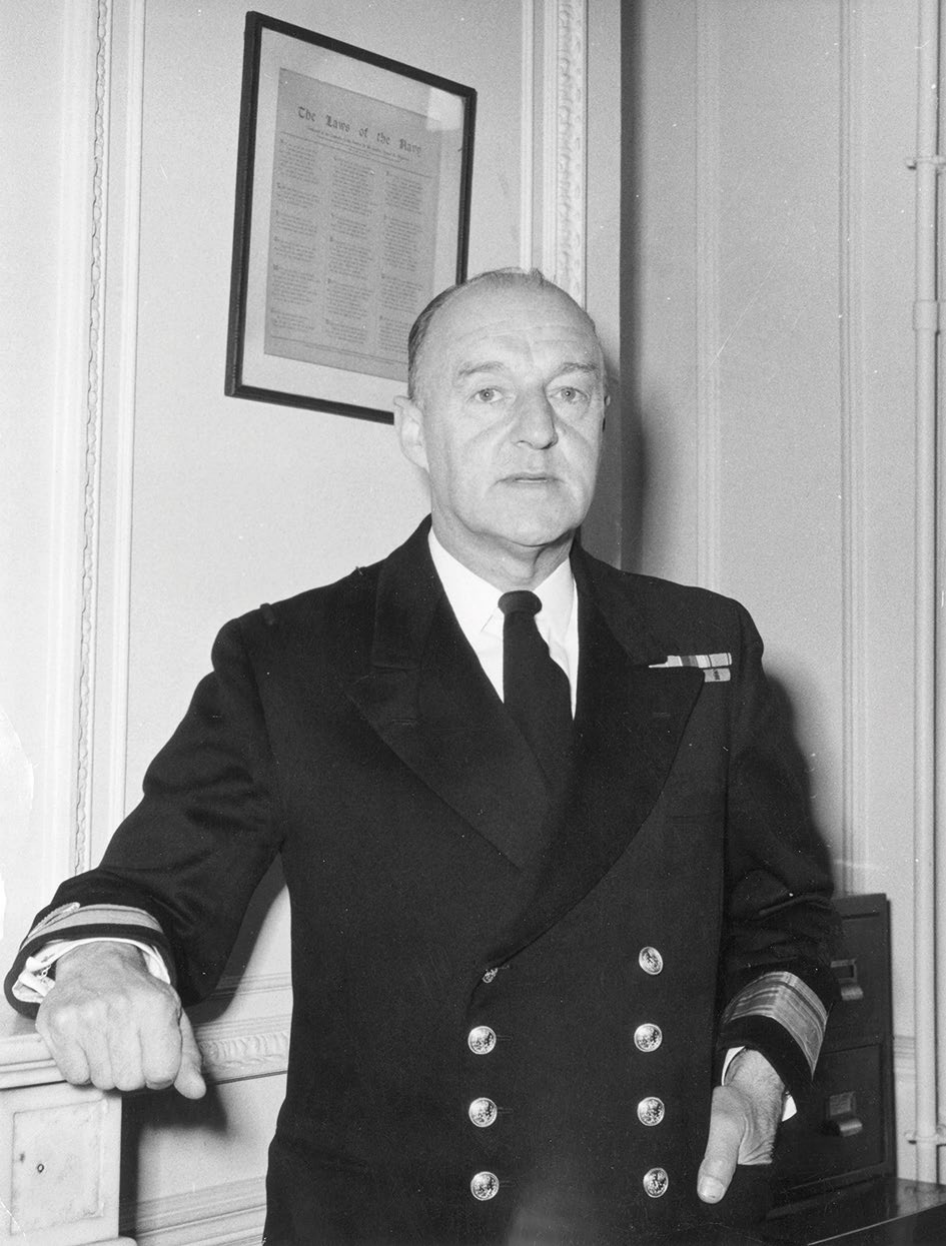
Rear Admiral W. Holgate CB, OBE, QHDS, LDS, FDSRCS Eng (reproduced with permission from the Imperial War Museum, London. ©Crown copyright reproduced under delegated authority from The Keeper of Public Records. Image: IWM [A 34504])

In 1949, Aneurin Bevan, Secretary of State for Health, used one of these Instruments delegated to him by Parliament in to set up four Standing Advisory Committees to guide him and the Central Council in matters affecting the four specialist areas of healthcare provision (medicine, dentistry, nursing and midwifery, and pharmacy) (Fig. 1).^5^ Members of the four Advisory Committees were appointed by the Secretary of State for Health on the advice of their Chief Professional Officers and it is known that for dentistry, the Chief Dental Officer at the time, Rear Admiral William Holgate (Fig. 2), also consulted senior members of the profession, as well as officers of the few specialist dental societies which existed at that time. Note also that at this time, in addition to the services shown in Figure 1, local authorities also provided school health services, which until 1974 were not part of the NHS. These included dental services for which the responsible Ministry was that of Education, not Health. However, the Chief Dental Officer was later given powers to inspect local authority dental services following adverse comments and doubtless liaised with the SDAC.

The duty of the SDAC and the three other Standing Committees was to advise the Minister and the Central Council as they felt fit upon such matters relating to the service with which the committee was concerned, and also provide advice on issues referred to them by the Minister or the Central Council.^6^ The SDAC met twice-yearly and its advice enabled the Minster to react quickly to meet the needs of the new NHS using his statutory powers.

## The SDAC Working Party on Orthodontics

By the 1980s, there was increasing concern among members of the five orthodontic societies in the United Kingdom (UK) who felt that the standard of the country's orthodontic treatment compared very poorly to that provided in other European countries.^7^ At this time, much of it was still being delivered in the General Dental Service (GDS) of the NHS by general practitioners using removable orthodontic appliances.^8^ However, 60% of GDS orthodontic treatment was now being delivered by dentists who had achieved a postgraduate orthodontic qualification, and were trained to use the full range of orthodontic techniques.^9^ Even so, only 10% of such cases were being treated using fixed appliances. Data supplied by the Dental Practice Board to the SDAC Working Party on Orthodontics in 1991 revealed that of the 9,108 dentists who provided some GDS orthodontic treatment in England and Wales, 240 held a postgraduate orthodontic qualification. Already by the early 1980s, calls were being made for these unregulated specialists to be identified and used appropriately.^10^^,^^11^^,^^12^ The development of the Peer Assessment Rating (PAR) Index in 1992 provided the profession with an objective means of assessing the standard of orthodontic treatment,^13^^,^^14^ and research using this Index, as well as patients' own reported dissatisfaction with the result of their treatment with removable appliances,^15^ now brought deficiencies in the standard of GDS orthodontics into sharp focus.

In 1989, just before he came to the end of his term on the Department of Health's Standing Dental Advisory Committee, Professor Norman Robertson, aware of these concerns and their significance for British orthodontics,^16^ ensured that the next SDAC would examine the provision and delivery of UK NHS orthodontic care as a whole. As a result, in April 1991, the new Committee gained approval from the Secretary of State for the funding of an Orthodontic Working Party. This was set up under the Chairmanship Ralph Followell (Box 1). Its terms of reference were ‘to consider the provision of NHS orthodontic care to patients receiving dental care within the NHS in England and Wales and to make recommendations about the objectives of care, the quality of care, value for money, and meeting patient's needs'.

The Working Party first met on 7 February 1991 and following an appeal for information by the Chairman, 128 submissions were received from organisations and individuals.^17^ In May 1992, the Working Party's 176-page fully costed report was presented to the SDAC.^18^ It contained much hitherto unpublished data as well as 89 references to published works. However, at the SDAC meeting on 19 May 1992, the Dental Public Health representatives on the Committee, led by Professor Roger Anderson of Birmingham, Past President of the British Society for the Study of Community Dentistry, argued successfully that the acceptance of the Report would run the risk of removing essential funding from existing overstretched basic dental services. His concerns were well-founded as, at that time, an overspend following the implementation of the new GDS contract was threatening a cut of 7% in GDS fees.^19^ As a result, the Working Party's Report was only ‘received' by the SDAC rather than ‘accepted', and thus was never published.

After the meeting in May 1992, the Chairman, Professor Murray's greatest concern was that from what he had learned, the UK treatment of cleft lip and palate was very poor and needed to be improved as a matter of urgency. The Clinical Standards Advisory Group (CSAG) had been set up in 1991 under the terms of the NHS and Community Care Act 1990 Section 2 to promote greater collaboration between health and social care agencies and enhance the quality of care provided.^20^ As Chairman of the SDAC, Professor Murray was an *ex officio* member of CSAG and was able to persuade the Group to set up a working party to advise on the current provisions for cleft care. The CSAG Working Party report was to have far reaching effects.^21^

Box 1 Members of the Standing Dental Advisory Committee Working Party on Orthodontics **Chairman**

- Mr R. Followell BDS, LDS RCS
  - General Dental Practitioner
  - Former Chairman of BDA Council and of its General Dental Services Committee

**Members**

- Mr D. DiBiase BDS, FDS, D Orth RCS
  - Consultant in Orthodontics, Southend Hospital
  - Chairman of the British Society for the Study of Orthodontics.
- Mr D. J. Birnie BDS, FDS, M Orth RCS
  - Consultant in Orthodontics, Queen Alexandra Hospital, Cosham
  - Past Secretary of the Consultant Orthodontists Group
- Mr R. A. Heesterman, BDS, DDH, LDS RCS
  - Specialist in Community Dental Health, Sheffield Health Authority
- Mr D. B. Lawton BDS, D.Orth RCS
  - Specialist Orthodontic Practitioner, Sevenoaks
  - Past Chairman of the British Orthodontic Association
- Mr D. W. Lester BChD, LDS RCS
  - General Dental Practitioner, Cuffley
  - Member of the BDA General Dental Services Committee
- Professor N. Robertson CBE, MDS, DDS, FDS, DDO
  - Dean of the Dental School, Cardiff
  - Past Chairman of the Association of University Teachers of Orthodontics
- Mrs M. H. Seward CBE, BDS, MDS, MCCD, FDS RCS
  - Member of the Standing Dental Advisory Committee
  - Member of the GDC
  - President Elect of the British Dental Association
- Mrs A. Smith
  - Policy and Development Officer, National Consumer Council, London
- Professor C. D. Stephens BDS, MDS, FDS, M Orth RCS
  - Professor of Child Dental Health, University of Bristol
  - Member, Standing Dental Advisory Committee

## Strategy or serendipity?

Despite its non-acceptance by the SDAC, 20 years later, all the main recommendations of the Working Party have either been implemented or are in hand (Box 2).^22^^,^^23^^,^^24^^,^^25^^,^^26^^,^^27^^,^^28^

There were several contributory reasons for this remarkable success. First, the carefully chosen composition of the Working Party ensured that all UK specialist and non-specialist dental interests were represented and were required to agree a report. Secondly, the publication of the *Schanshieff Report on Unnecessary Dentistry* in 1986,^29^ while its conclusions on NHS orthodontic treatment were shown to be unsoundly based and were subsequently comprehensively rejected by the British Dental Association, had made the five UK orthodontic societies realise they needed to resolve their differences and in future speak with one voice. To this end, by 1992, negotiations were already well in hand which led to the establishment of the unified British Orthodontic Society two years later.^12^ Finally, the expectation that the Working Party's report was likely to be brought to the attention of the Department of Health ensured that its content and recommendations were carefully reasoned and supported by references to all the latest research.

Box 2 The dates of implementation of the Working Party's 1992 recommendations **1998**

- Establishment of a UK specialist list for orthodontics

**2002**

- Introduction of orthodontic therapists

**2005**

- Restructuring of cleft lip and palate services
- Use of Indices of orthodontic treatment need
- Use of Peer Assessment Rating (PAR) index to monitor General Dental Service orthodontic treatment standards

**2018**

- Orthodontic manpower planning

## Discussion

It was entirely appropriate in 1948 for Parliament to grant the Minister of Health statutory powers to introduce urgent measures essential for establishing the new and untested system of social healthcare provision.^2^ Among the most visionary outcomes of this legislation was the creation in 1949 of four Standing Advisory Health Committees, including the SDAC. These bodies provided independent, professional advice directly to the Minister – advice that was not filtered or diluted through the civil service. This direct line of communication was efficient and democratic, allowing ministers to make informed decisions based on unvarnished clinical expertise. It also allowed advisory committees to raise issues of concern unprompted – a strength that allowed Professor Norman Robertson, through the SDAC, to initiate a report that, while unpublished, led to far-reaching improvements in UK orthodontic care. However, in 2008, the then Secretary of State for Health, Alan Johnson, abolished the SDAC using the same delegated powers that had established it.^30^ This followed a 2007 Department of Health report (devoid of supporting evidence) claiming that *ad hoc,* subject-specific committees were more ‘cost-effective' and that the costs of supporting the SDAC were ‘disproportionate to its outputs'. This rationale was questionable at best, as the Committee's operations involved only secretarial support and reimbursed travel expenses for biennial meetings. Sadly, in 2010, the House of Lords, reviewing a raft of similar enactments made under Statutory Instruments, saw no reason to question this decision.^31^ Since its abolition, no clear replacement for the SDAC has emerged, and it remains unclear whether any structured, independent dental advisory body has been re-established within the Department of Health. In the absence of such a body, the UK has seen major gaps in the oversight and development of dental policy. The Department's claim that there were insufficient issues to warrant the committee's continuation now rings hollow. If the SDAC had still existed, one must ask: would we still be waiting for the long-overdue ten-year review of children's dental health in England and Wales? Would the current access crisis, where nine out of ten NHS dental practices are reportedly not accepting new adult patients,^32^ have escalated to this point? These questions gained further urgency during a recent parliamentary review of NHS dentistry in England, where Chief Medical Officer, Chris Whitty made the startling admission that the substantial public funds allocated to improve access to NHS dentistry had made ‘little impact'. This public confession, coming from the nation's most senior medical advisor, was both embarrassing and revealing. It underscored a fundamental policy failure: decisions were made without credible, continuous and independent professional guidance. One cannot help but wonder whether such a situation would have occurred if a body like the SDAC still existed. Would an independent voice, with statutory authority and direct access to the Minister, have flagged early signs of dysfunction in the dental contract system? Would it have advocated for reforms grounded in clinical reality rather than bureaucratic expediency? The original purpose of the SDAC was not simply to respond to ministerial requests but to bring pressing issues to light, long before they escalated into national crises. Its abolition and the failure to establish a credible replacement have left a vacuum in Central Government. The consequence is a health policy environment increasingly reactive rather than preventive, guided more by short-term politics than long-term public interest.

## Conclusion

In conclusion, the importance of maintaining independent advisory voices within Central Government cannot be overstated. Ministers are deprived of the full picture without them and the public suffers the consequences. Reinstating a statutory, professional advisory committee for dentistry would be a meaningful step toward restoring credibility, transparency and effectiveness in oral health policy.

## References

[1] UK Government. *The National Health Service Act 1946: 9 & 10 Geo. 6, ch.81, Part I, Section 2*. 1946.

[2] Hill C, Woodcock J. *The National Health Service*. Appendix III. London: Christopher Johnson, 1949.

[3] Stephens C D. Frederick Ballard – tireless champion of the NHS. *Dent Hist* 2018; **63:** 45–51.

[4] UK Government. *The Statutory Instruments Act 1946: 9 & 10 Geo. 6. ch. 36*. 1946.

[5] UK Government. The National Health Service (Central Health Service Council and Standing Advisory Committees) Regulation 1948. Available at https://www.legislation.gov.uk/uksi/2010/635/contents/made (accessed 11 May 2025).

[6] Speller S R. *The National Health Service Act 1946.* London: H. K. Lewis, 1948.

[7] Mills J R, Leighton B C, Cousins A J *et al*. Working Party on British Orthodontic Standards. *Br J Orthod* 1980; **7:** 103–107. 10.1179/bjo.7.2.103 6932966

[8] Shaw W C. Improving British orthodontic standards. *Br Dent J* 1983; **155:** 133–135. 10.1038/sj.bdj.4805153 6578817

[9] UK Parliament. *Report of the Dental Strategy Review Group: Towards Better Dental Health – Guidelines for the Future.* London: Department of Health and Social Security, 1981.

[10] O'Brien K D, Corkhill C M. The specialist orthodontic practitioner – the 1989 survey. *Br Dent J* 1990; **168:** 471–475. 10.1038/sj.bdj.4807245 2369542

[11] Usiskin L. The orthodontist in the GDS. *Br Dent J* 1982; **153:** 50–51. 10.1038/sj.bdj.4804850 6956353

[12] Stephens C D. The unification of UK orthodontic societies 1970–1994. *Dent Hist* 2021; **66:** 17–24.

[13] Richmond S, Shaw W C, Roberts C T, Andrews M. The PAR Index (Peer Assessment Rating): methods to determine outcome of orthodontic treatment in terms of improvement in standards. *Euro J Orthod* 1992; **14:** 180–187. 10.1093/ejo/14.3.180 1628684

[14] Richmond S, Shaw W C, O'Brien K D *et al*. The development of the PAR Index (Peer Assessment Rating): reliability and validity. *Europ J Orthod* 1992; **14:** 125–139. 10.1093/ejo/14.2.125 1582457

[15] Gravely J F. Who should practise orthodontics? *Br J Orthod* 1989; **16:** 235–241. 10.1179/bjo.16.4.235 2818999

[16] Robertson N R, Hoyle B A. Orthodontic treatment – time for a change. *Br J Orthod* 1983; **10:** 154–156. 10.1179/bjo.10.3.154 6575824

[17] Followell R. SDAC Orthodontic Review. *Br Dent J* 1991; **170:** 285.

[18] Standing Dental Advisory Committee of the Department of Health*. Orthodontics in the National Health Service – Report of an expert working party of the Standing Dental Advisory Committee of the Department of Health*. 1992. BDA Library ref. RBR XX FOL.

[19] British Dental Journal. Unanimous vote at LDS Conference against GDS fee cut. *Br Dent J* 1992; **172:** 295.

[20] Higginson G. Functions of the Clinical Standards Advisory Group. *BMJ* 1992; **304:** 571. 10.1136/bmj.304.6826.571 PMC1881425 1559070

[21] Fitzsimons K J, Mukarram S, Copley L P, Deacon S A, van der Meulen J H. Centralisation of services for children with cleft lip or palate in England: a study of hospital episode statistics. *BMC Health Serv Res* 2012; **12:** 148. 10.1186/1472-6963-12-148 PMC3464162 22682355

[22] UK Government. *The European Primary, Specialist Dental Qualifications Regulations 1998*. 1998.

[23] Kettler C J, Stephens C D, Riches R. Moves to establish orthodontic therapists in the UK (1967–2005). *Dent Hist* 2022; **67:** 44–50.

[24] General Dental Council. Preparing for Practice. 2015. Available at https://www.gdc-uk.org/docs/default-source/education-and-cpd/preparing-for-practice-(revised-2015).pdf?sfvrsn=81d58c49_2 (accessed 1 January 2026).

[25] NHS England. NHS Standard Contract for cleft lip and/or palate services including velopharyngeal dysfunction (all ages). 2013. Available at https://www.england.nhs.uk/wp-content/uploads/2013/06/d07-cleft-lip.pdf (accessed 1 January 2026).

[26] UK Government. The National Health Service (General Dental Services Contract) Regulations 2005. Available at https://www.legislation.gov.uk/uksi/2005/3361/pdfs/uksi_20053361_en.pdf (accessed 27 May 2025).

[27] NHS England. Assessment of orthodontic treatment need and level of service provision for the resident population of Cheshire and Merseyside 2018. 2018. Available at https://www.england.nhs.uk/north/wp-content/uploads/sites/5/2018/09/CM-Orthodontic-Needs-Assessment.pdf (accessed 1 January 2026).

[28] Deeming G, Cobourne M T. Making sense in a complex world of orthodontic commissioning. *J Orthod* 2021; **48:** 455–457. 10.1177/14653125211059621 34873948

[29] UK Parliament. *Report of the Committee of Enquiry into Unnecessary Dental Treatment*. 1987.

[30] UK Government*. The National Health Service (Standing Advisory Committees) Amendment Order 2010*. 2010.

[31] House of Lords. *Merits of Statutory Instruments Committee – Fifteenth Report*. 2010.

[32] Green R, Agerholm H, Rogers L. Full extent of NHS dentistry shortage revealed by far-reaching BBC research. *BBC News* (London) 8 August 2022.

